# Supplementary material for: The EIF4EBP1 gene encoding 4EBP1 is transcriptionally upregulated by MYC and linked to shorter survival in medulloblastoma
Source: Cell Death Discov. 2025 Jul 16;11:330. doi: 10.1038/s41420-025-02601-x (PMC12267489; doi:10.1038/s41420-025-02601-x)
Supplement: Supplementary file 1 — Supplementary information [file 41420_2025_2601_MOESM1_ESM.docx]

**Article title:** The *EIF4EBP1* gene encoding 4EBP1 is transcriptionally upregulated by MYC and linked to shorter survival in medulloblastoma

**Journal name:** Cell Death and Discovery

**Author names:**

Laura Hruby^1*^, Katerina Schaal^2*^, Alberto Delaidelli^3,4^, Daniel Picard^1,2,5,6^, Christopher Dunham^4^, Oksana Lewandowska^1,7^, Tobias Reiff^7^, Magalie Larcher^8^, Celio Pouponnot^8^, Poul HB Sorensen^3,4^, Barak Rotblat^9,10^, Guido Reifenberger^1,5^, Marc Remke^1,2,5,6^, Gabriel Leprivier^1^

**Affiliations:**

^1^Institute of Neuropathology, University Hospital Düsseldorf and Medical Faculty, Heinrich Heine University, Düsseldorf, Germany.

^2^Department of Pediatric Oncology, Hematology, and Clinical Immunology, University Hospital Düsseldorf and Medical Faculty, Heinrich Heine University, Düsseldorf, Germany.

^3^Department of Molecular Oncology, British Columbia Cancer Research Centre, Vancouver, BC, Canada.

^4^Department of Pathology and Laboratory Medicine, University of British Columbia, Vancouver, BC, Canada.

^5^German Cancer Consortium (DKTK), Partner site Essen/Düsseldorf, Düsseldorf, Germany.

^6^Department of Pediatric Oncology and Hematology, Saarland University Medical Center and Saarland University Faculty of Medicine, Homburg/Saar, Germany.

^7^Institute of Genetics, Heinrich Heine University, Düsseldorf, Germany.

^8^Institut Curie, Université Paris-Sud, Université Paris-Saclay, CNRS UMR 3347, INSERM U1021, Orsay, Paris, France.

^9^Department of Life Sciences, Faculty of Natural Sciences, Ben-Gurion University of the Negev, Beer-Sheva, Israel.

^10^The National Institute for Biotechnology in the Negev, Ben-Gurion University of the Negev, Beer-Sheva, Israel.

**Correspondence to:** [gabriel.leprivier@med.uni-duesseldorf.de](mailto:gabriel.leprivier@med.uni-duesseldorf.de).

**Supplementary figure 1*.* Analyses of *EIF4EBP1* expression levels and *EIF4EBP1* and *MYC(N)* co-expression levels in MB groups and subgroups.**

**A**, DNA methylation levels of 18 CpG sites located within the *EIF4EBP1* promoter region (human genome GRCh 37/hg19; Chr8: 37,886,955-37,917,868) using the Chatterton dataset for fetal brain (FB) (n=9) [1] and the Cavalli *et al.* dataset for MB tissues (n=763) [2] with 0 representing unmethylated and 1 representing fully methylated CpG sites. A two-tailed Fisher’s exact test was used to determine statistical differences between FB and MB samples.

**B**, Expression levels of *EIF4EBP1* mRNA in primary and relapse MB tissues from the Pomeroy [3] cohort.

**C and D**, Expression levels of *EIF4EBP1* mRNA in primary and metastatic tissues pooled from the Delattre, Gilbertson [4] and Thompson cohorts (microarray up133p2) (C) and from the Cavalli *et al.* cohort (microarray hugene11t) [2] (D).

**E and F**, Expression levels of *EIF4EBP1* levels in Group 3 and Group 4 MBs of the Northcott *et al.* cohort [5] (E) or according to Group 4 MB subgroups of the Cavalli *et al.* cohort [2] (F).

Significance in B-F was calculated using an unpaired and two-tailed parametric t-test (**p<0.01, ***p<0.001, ****p<0.0001).

**G and H**, Expression levels of *EIF4EBP1* mRNA according to *EIF4EBP1* copy number variation in Group 3 (G) and Group 4 (H) MB subgroups alpha, beta and gamma from the Cavalli *et al.* cohort [2] categorized as *EIF4EBP1* copy number loss (hemizygous deletion [loss]), *EIF4EBP1* neutral copy number (neutral), or *EIF4EBP1* low-level copy number gain (gain).

**I-L**, Expression levels of *EIF4EBP1* mRNA in MB patient samples plotted against the mRNA expression levels of *MYC* (I and J) or *MYCN* (K and L) in Group 4 MBs using the Cavalli *et al.* [2] and Pfister [6] cohorts as indicated. Co-expression levels were quantified by calculating the Pearson correlation coefficient.


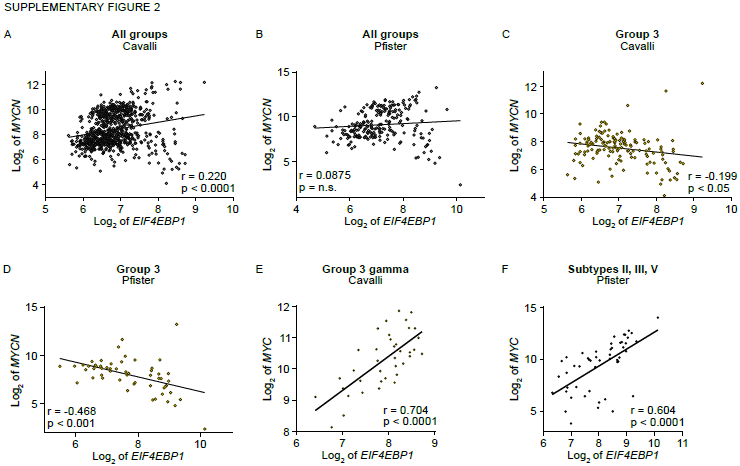


**Supplementary figure 2. Analyses of *EIF4EBP1* and *MYC(N)* co-expression levels in MB groups and subgroups.**

**A-D,** Expression levels of *EIF4EBP1* mRNA in MB patient samples plotted against the mRNA expression levels of *MYCN* in all patients (A and B) and in Group 3 MB patients (C and D) using the Cavalli *et al.* [2] and Pfister [6] cohorts as indicated. Co-expression levels were quantified by calculating the Pearson correlation coefficient.

**E and F,** Expression levels of *EIF4EBP1* mRNA in MB patient samples plotted against the mRNA expression levels of *MYC* in Group 3 MB gamma (E) and Heidelberg MB subtypes II, III and V (F) using the Cavalli *et al.* [2] and the Pfister [6] cohorts as indicated (see Table S2 for the number of patient samples per group). Co-expression levels were quantified by calculating the Pearson correlation coefficient.

**Supplementary figure 3. *EIF4EBP1* mRNA expression levels do not correlate with overall survival in Group 4 MB and SHH MB patients.**

**A-C**, Kaplan-Meier survival estimates of overall survival of MB patients stratified by their *EIF4EBP1* mRNA expression levels in Group 4 (A and B) and in SHH (C) using the Cavalli *et al.* [2] and Pomeroy [3] cohorts as indicated. The data were obtained from R2 Genomics and visualization platform and the median expression level of *EIF4EBP1* mRNA was used as cut-off. Significance was calculated with the log-rank test.

**Supplementary figure 4. 4EBP1 protein expression is upregulated in MB tissue samples from relapsed compared to primary tumors.**

**A**, Primary and relapsed MB tissue samples were immunostained using an anti-4EP1 antibody. Staining intensity was plotted and significance was calculated using an unpaired and two-tailed parametric t-test (**p < 0.01).

**B,** Representative immunostaining of WNT, SHH, Group 3 and Group 4 MB tissue samples using an anti-4EP1 antibody.

**Supplementary figure 5. Impact of MYC knock down on rates of cell death and proliferation in Med8A MB cell line.**

**A and B**, Med8A MB cells were transiently transfected with negative control (siCtrl) or a pool of four different siRNAs (see Table S5) targeting MYC (siMYC) and cells were grown for 72 h. Cell death was measured using PI and flow cytometry (A). Proliferation was measured using an EdU Assay (B). Data represent the mean ± SD. Significance was calculated using an unpaired and two-tailed parametric t-test (*p<0.05).

**Supplementary figure 6. Impact of 4EBP1 knock down on migratory capacities and energy levels of MB cells.**

**A and B**, Control (ishCtrl) or stable inducible 4EBP1 knock down (ish4EBP1#1 and #2) Med8A (A) or HD-MB03 (B) were treated with 1 µg/ml doxycycline for 48 h. 50,000 cells were plated per well around a cell stopper. After 24 h, cell stoppers were removed and cells were allowed to migrate into the empty space for 72 h. Migrated cells were stained with Hoechst and counted using ImageJ. Data represent the mean ± SD of three individual replicates. Significance was calculated using an unpaired and two-tailed parametric t-test (*p<0.05).
**C and D**, Control (ishCtrl) or stable inducible 4EBP1 knock down (ish4EBP1#1 and #2) Med8A (C) or HD-MB03 (D) were treated with 1 µg/ml doxycycline for 48 h. ATP levels were assessed using CellTiter-Glo® Luminescent Cell Viability Assay. Data represent the mean ± SD of three individual replicates. Significance was calculated using an unpaired and two-tailed parametric t-test (*p<0.05).

**Supplementary table 1: Overview of the analyzed non-neoplastic brain tissue and medulloblastoma cohorts.**

| **Tissue / MB cohorts** | **Cohort name** | **Microarray** | **GEO ID / PUB med link** | **References** |
| --- | --- | --- | --- | --- |
| **Non-neoplastic brain tissue** | Normal cerebellum - “Roth *et al."* | u133p2 | GSE3526 | [7] |
|  | Mixed Medulloblastoma public - "Pomeroy” | u133p2 | 21098324 | [3] |
|  | | | | |
| **Medulloblastoma** | Tumor Medulloblastoma public - "Delattre | u133p2 | Information not available |  |
|  | Tumor Medulloblastoma Ependymoma - "denBoer“ | u133p2 | GSE74195 | [8] |
|  | Tumor Medulloblastoma - "Gilbertson“ | u133p2 | GSE37418 | [4] |
|  | Tumor Medulloblastoma - ATRT - "Hsieh“ | u133p2 | GSE67851 | [9] |
|  | Tumor Medulloblastoma PLoS One - "Kool *et al."* | u133p2 | GSE10327 | [10] |
|  | Tumor Medulloblastoma - "Pfister“ | u133p2 | 28726821 | [6] |
|  | Mixed Medulloblastoma public - "Pomeroy” | u133a | 21098324 | [3] |
|  | Tumor Medulloblastoma - "Thompson” | u133a | Information not available |  |
|  | Tumor Medulloblastoma - "Cavalli *et al."* | hugene11t | GSE85217 | [2] |
|  | Tumor Medulloblastoma MAGIC - "Northcott *et al."* | hugene11t | GSE37382 | [5] |

**Supplementary table 2: Overview of the number of patients per subgroup in the different cohorts.**

| **MB cohorts** | **MB group** | **Number of patients** |
| --- | --- | --- |
| **"Cavalli** ***et al."*** | **All MB groups** | **763** |
|  | SHH | 223 |
|  | WNT | 70 |
|  | Group 3 | 144 |
|  | Group 4 | 326 |
| **"Gilbertson”** | SHH | 10 |
|  | WNT | 8 |
|  | Group 3 | 16 |
|  | Group 4 | 39 |
| **"Kool *et al."*** | SHH | 15 |
|  | WNT | 9 |
|  | Group 3 | 11 |
|  | Group 4 | 27 |
| **"Northcott *et al."*** | Group 3 | 46 |
|  | Group 4 | 188 |
| **"Pfister”** | All MB groups | 223 |
|  | SHH | 59 |
|  | WNT | 17 |
|  | Group 3 | 56 |
|  | Group 4 | 91 |
| **"Pomeroy”** | SHH | 52 |
|  | WNT | 14 |
|  | Group 3 | 51 |
|  | Group 4 | 71 |

**Supplementary table 3: Cell lines used for the analyzed ChIP-seq data of MYC.**

| **UCSC Accession** | **Cell line** |
| --- | --- |
| EH001867 | K562 |
| EH002800 | K562 |
| EH000670 | K562 |
| EH003436 | MCF-7 |
| EH001807 | NB4 |
| EH000547 | GM12878 |
| EH000545 | HepG2 |
| EH002795 | H1-hESC |
| EH000596 | H1-hESC |

**Supplementary table 4: Chromosomal positions of the CpG sites within the *EIF4EBP1* promoter region Chr8: 37,886,955-37,917,868 (human genome GRCh 37/hg19).**

| **CpG site (ID)** | **Chromosomal location** |
| --- | --- |
| cg00354863 | Chr8:37886955 |
| cg22545112 | Chr8:37887635 |
| cg22728323 | Chr8:37887713 |
| cg09510263 | Chr8:37887715 |
| cg21815479 | Chr8:37887900 |
| cg09188300 | Chr8:37887903 |
| cg04423064 | Chr8:37887926 |
| cg05695311 | Chr8:37887949 |
| cg18350458 | Chr8:37887952 |
| cg13426096 | Chr8:37887990 |
| cg18834416 | Chr8:37888184 |
| cg25500285 | Chr8:37888493 |
| cg03613132 | Chr8:37888764 |
| cg04217082 | Chr8:37889752 |
| cg17887364 | Chr8:37891957 |
| cg02138124 | Chr8:37901697 |
| cg06437703 | Chr8:37914619 |
| cg26921611 | Chr8:37917868 |

**Supplementary table 5: List of siRNA sequences.**

| **Target gene and siRNA name** | **siRNA sequence** |
| --- | --- |
| Dharmacon | |
| Non-targeting | 5’- UAAGGCUAUGAAGAGAUAC -3’ |
|  | 5’- AUGUAUUGGCCUGUAUUAG -3’ |
|  | 5’- AUGAACGUGAAUUGCUCAA -3’ |
|  | 5’- UGGUUUACAUGUCGACUAA -3’ |
| MYC si14 | 5’- AACGUUAGCUUCACCAACA -3’ |
| MYC si35 | 5’- CUACCAGGCUGCGCGCAAA -3’ |
| MYC SMART pool | 5’- ACGGAACUCUUGUGCGUAA -3’ |
|  | 5’- GAACACACAACGUCUUGGA -3’ |
|  | 5’- AACGUUAGCUUCACCAACA -3’ |
|  | 5’- CGAUGUUGUUUCUGUGGAA -3’ |

**Supplementary table 6: List of RT-qPCR primer sequences.**

| **Target transcript** | **Primer sequence** |
| --- | --- |
| *4EBP1* | FW: 5’-AGCCCTTCCAGTGATGAGC-3’  RV: 5’-TGTCCATCTCAAACTGTGACTCTT-3’ |
| *GusB* | FW: 5’-GTTTTTGATCCAGACCCAGATG-3’  RV: 5’-GCCCATTATTCAGAGCGAGTA-3’ |
| *MYC* | FW: 5’-GTCAAGAAGCGAACACACAAC-3’  RV: 5’-TTGGACGGACAGGATGTATGC-3’ |
| *PPIA* | FW: 5’-TTATTTGGGTTGCTCCCTTC-3’  RV: 5’-AAGTGTGCCAAATCTGCAAG-3’ |
| *ACTB* | FW: 5’-TCCCCCAACTTGAGATGTATG-3’  RV: 5’-ACTGGTCTCAAGTCAGTGTACAGG-3’ |

**Supplementary table 7: List of antibodies used for immunoblots.**

| **Antibody** | **Company** | **Catalog number** |
| --- | --- | --- |
| 4EBP1 (53H11) | Cell signaling, Cambridge, UK | #9644S |
| Anti-rabbit IgG, HRP linked antibody | Cell signaling | #7074 |
| GAPDH (14C10) | Cell signaling | #2118S |
| IRDye® 800CW Goat anti-Mouse IgG Secondary Antibody | LI-COR Bioscience, Bad Homburg, Germany | #925-32210 |
| IRDye® 800CW Goat anti-Rabbit IgG Secondary Antibody | LI-COR Bioscience | #925-32211 |
| MYC | Cell signaling | #5605 |
| $\text{β}$-ACTIN | Sigma Aldrich, St Louis, USA | #A2228 |

**Supplementary table 8: Results of pair-wise comparison of *EIF4EBP1* expression levels between different MB groups in the investigated cohorts (corresponding to Fig. 1B and C).**

| **MB Cohort** | **MB group comparisons** | **Significance** |
| --- | --- | --- |
| **“Cavalli *et al.*”** | Group 3 vs. SHH | ** |
|  | Group 3 vs. WNT | n.s. |
|  | Group 3 vs. Group 4 | **** |
|  | WNT vs. SHH | **** |
|  | WNT vs. Group 4 | **** |
|  | SHH vs. Group 4 | **** |
|  | | |
| **Cohort pool**  **(Pfister, Gilbertson, Pomeroy, Kool *et al.*)** | Group 3 vs. SHH | n.s. |
|  | Group 3 vs. WNT | n.s. |
|  | Group 3 vs. Group 4 | **** |
|  | WNT vs. SHH | n.s. |
|  | WNT vs. Group 4 | **** |
|  | SHH vs. Group 4 | **** |

**REFERENCES**

1 Chatterton Z, Hartley BJ, Seok MH, Mendelev N, Chen S, Milekic M et al. In utero exposure to maternal smoking is associated with DNA methylation alterations and reduced neuronal content in the developing fetal brain. Epigenetics Chromatin. 2017;10:4. <https://doi.org:10.1186/s13072-017-0111-y>

2 Cavalli FMG, Remke M, Rampasek L, Peacock J, Shih DJH, Luu B et al. Intertumoral Heterogeneity within Medulloblastoma Subgroups. Cancer Cell. 2017;31:737-754 e736. <https://doi.org:10.1016/j.ccell.2017.05.005>

3 Cho YJ, Tsherniak A, Tamayo P, Santagata S, Ligon A, Greulich H et al. Integrative genomic analysis of medulloblastoma identifies a molecular subgroup that drives poor clinical outcome. Journal of Clinical Oncology. 2011;29:1424-1430. <https://doi.org:10.1200/JCO.2010.28.5148>

4 Robinson G, Parker M, Kranenburg TA, Lu C, Chen X, Ding L et al. Novel mutations target distinct subgroups of medulloblastoma. Nature. 2012;488:43-48. <https://doi.org:10.1038/nature11213>

5 Northcott PA, Shih DJ, Peacock J, Garzia L, Morrissy AS, Zichner T et al. Subgroup-specific structural variation across 1,000 medulloblastoma genomes. Nature. 2012;488:49-56. <https://doi.org:10.1038/nature11327>

6 Northcott PA, Buchhalter I, Morrissy AS, Hovestadt V, Weischenfeldt J, Ehrenberger T et al. The whole-genome landscape of medulloblastoma subtypes. Nature. 2017;547:311-317. <https://doi.org:10.1038/nature22973>

7 Roth RB, Hevezi P, Lee J, Willhite D, Lechner SM, Foster AC et al. Gene expression analyses reveal molecular relationships among 20 regions of the human CNS. Neurogenetics. 2006;7:67-80. <https://doi.org:10.1007/s10048-006-0032-6>

8 de Bont JM, Kros JM, Passier MM, Reddingius RE, Sillevis Smitt PA, Luider TM et al. Differential expression and prognostic significance of SOX genes in pediatric medulloblastoma and ependymoma identified by microarray analysis. Neuro Oncol. 2008;10:648-660. <https://doi.org:10.1215/15228517-2008-032>

9 Ho DM, Shih CC, Liang ML, Tsai CY, Hsieh TH, Tsai CH et al. Integrated genomics has identified a new AT/RT-like yet INI1-positive brain tumor subtype among primary pediatric embryonal tumors. BMC Med Genomics. 2015;8:32. <https://doi.org:10.1186/s12920-015-0103-3>

10 Kool M, Koster J, Bunt J, Hasselt NE, Lakeman A, van Sluis P et al. Integrated genomics identifies five medulloblastoma subtypes with distinct genetic profiles, pathway signatures and clinicopathological features. PLoS One. 2008;3:e3088. <https://doi.org:10.1371/journal.pone.0003088>
