# Supplementary figures and images for: The EIF4EBP1 gene encoding 4EBP1 is transcriptionally upregulated by MYC and linked to shorter survival in medulloblastoma
Source: Cell Death Discov. 2025 Jul 16;11:330. doi: 10.1038/s41420-025-02601-x (PMC12267489; doi:10.1038/s41420-025-02601-x)

**4B**

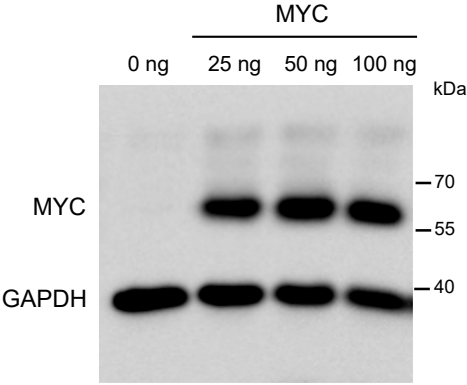

**4D**

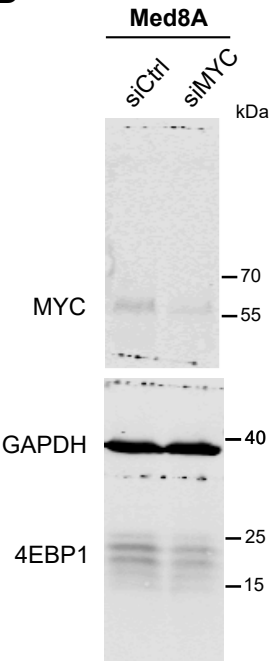

**4H**

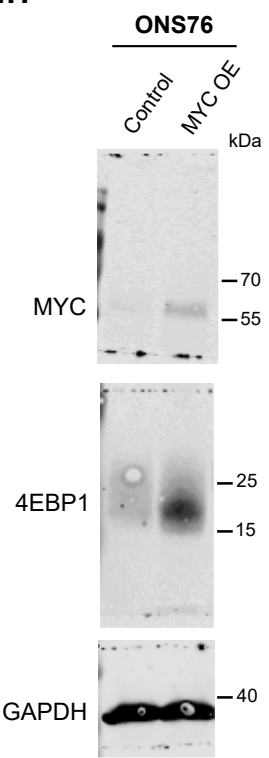

**4I**

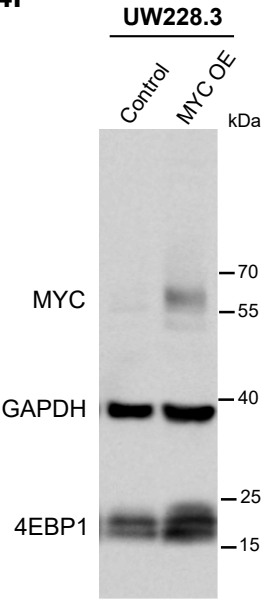

**5A**

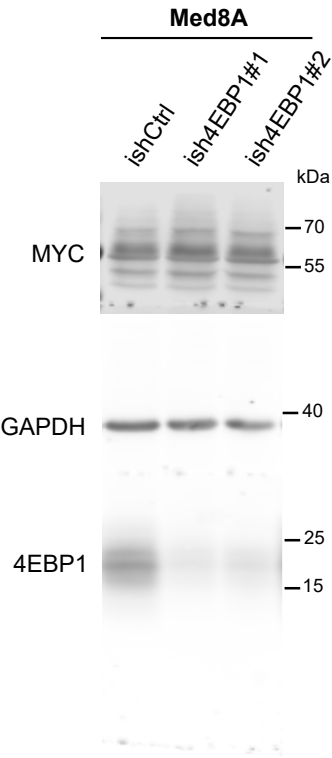

**5B**

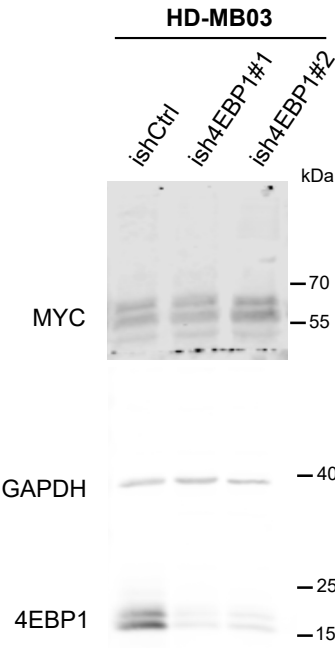

Supplement: Supplementary file 2 — Original blots [file 41420_2025_2601_MOESM2_ESM.pdf]
